# The complete chloroplast genome sequence of *Cinnamomum septentrionale* (Lauraceae) from Sichuan Province, China, a medicinal plant and phylogenetic analysis

**DOI:** 10.1080/23802359.2021.1967812

**Published:** 2021-08-24

**Authors:** Qin Lv, Bianjun Liang, Xiaojuan Guo

**Affiliations:** a Department of Chinese Medicine, NanYang Medical College, Nanyang, China; b Zhang Zhongjing College of Chinese Medicine, NanYang Institute of Technolog, Nanyang, China

**Keywords:** *Cinnamomum septentrionale*, chloroplast genome, phylogenetic analysis

## Abstract

*Cinnamomum septentrionale* is a commercially important timber tree and wild spice plant classified in the Lauraceae. Here we sequenced the complete plastid genome of *C. septentrionale* to contribute to its phylogenetics and bioinformatics. The chloroplast genome of *C. septentrionale* is 152,729 bp in length and includes a large single copy (LSC) region of 93,639 bp, a small single copy (SSC) region of 18,858 bp, and two inverted repeat (IR) regions of 20,114 bp. The GC content of LSC, SSC, IR and the whole genome is 37.9%, 33.8%, 44.4%, and 39.1%, respectively. There are 128 genes, including 84 protein-coding, 36 tRNA, and eight rRNA genes. The phylogenetic analysis resolved *C. septentrionale* in a sister position to *C. glanduliferum*. The complete chloroplast genome sequence of *C. septentrionale* provides valuable genomic information for future systematic studies on *Cinnamomum*.

*Cinnamomum septentrionale* Hand-Mazz, a large-sized evergreen tree species, is widely distributed in Guizhou, Sichuan, and Yunnan of SW China (http://foc.iplant.cn/). The essential oil isolated from the bark and leaves of *C. septentrionale* is rich in eucalyptol (Taha and Eldahshan 2017) and thus represent an important aromatic plant species in the genus *Cinnamomum* Trew (Kumar and Kumari 2019). For a better understanding of the relationships of *C. septentrionale* and other *Cinnamomum* species, it is necessary to reconstruct a phylogenetic tree based on high-throughput sequencing approaches. In the present study, we report and characterize the complete chloroplast (cp) genome of *C. septentrionale* based on Illumina pair-end sequencing and compare it to other genome sequences from Lauraceae. The results provide valuable genetic information for evolutionary and conservation studies of *C. septentrionale*.

The voucher specimen of *C. septentrionale* was collected from Nanchong, Sichuan province, China (106°08′E; 30°79′N) and is deposited at the herbarium of NanYang Medical College, Department of Chinese Medicine (http://www.nymc.edu.cn/, Lv Qin, 2931084169@qq.com) under the voucher number YM001. The DNA sample is stored at the Key Laboratory of Department of Chinese Medicine, Nanyang, China. Total genomic DNA was extracted using the DNA Secure Plant Kit (Tiangen Biotech, Beijing, China) following the manufacturer’s protocol. Library preparation and genomic sequencing on the Illumina Hiseq 2500 platform were conducted by Benagen (Benagen Inc., Wuhan, China). The raw sequence data have been deposited into NCBI SRA with project accession of SRR14793489. The raw data was filtered using Trimmomatic Version 0.32 with the default settings (Bolger et al. 2014). The filtered output yielded 5.4 Gb of raw 150 bp paired-end data. The obtained paired-end reads were assembled using SPAdes v.3.9.0 (Nurk et al. 2017). The assembled sequence was annotated in MPI-MP CHLOROBOX (https://chlorobox.mpimp-golm.mpg.de/geseq.html) via GeSeq using the reference cp genome of *C. glanduliferum* (MW369062, Zhao et al. 2019), and corrected using Geneious Prime v2020.2 (Kearse et al. 2012). Finally, the complete chloroplast genome of *C. septentrionale* was submitted to GenBank (Accession No. MZ128522).

The chloroplast genome of *C. septentrionale* is 152,729 bp in length and includes a large single copy (LSC) region of 93,639 bp, a small single copy (SSC) region of 18,858 bp, and two inverted repeat (IR) regions of 20,114 bp. The GC contents of LSC, SSC, IR and whole genome are 37.9%, 33.8%, 44.4%, and 39.1%, respectively. The chloroplast genome encodes 128 genes, including 84 protein-coding, 36 tRNA, and eight rRNA genes. The plastome of *C. septentrionale* was 14 bp and 10 bp larger than that of *C. glanduliferum* (MW369062) and *C. bodinieri* (152,719 bp, MH394415). It was also 31 bp and 46 bp smaller than that of *C. parthenoxylon* (152,760 bp, MH050971) and *C. burmanni* (152,775 bp, LAU00110). The overall GC content of *C. septentrionale* is consistent with *C. glanduliferum* (MW369062), *C. bodinieri* (MH394415) and *C. parthenoxylon* (MH050971), which are all 39.1%.

To further investigate the systematics of *C. septentrionale*, a Maximum-likelihood (ML) tree was constructed using complete chloroplast genome sequences of 12 other related species aligned with MAFFT (Katoh and Standley 2013). The analysis was performed using MEGA 7.0 (Kumar et al. 2016) with 1000 bootstrap replicates. The program operating parameters were set as follows: a Tamura 3-parameter (T92) nucleotide substitution model with 1000 bootstrap repetitions, accompanied by Gamma distributed with Invariant site (G + I) rates, and partial deletion of gaps/missing data. The phylogenetic analysis revealed that *C. septentrionale* was most related to *C. glanduliferum* as a sister group with 99% bootstrap support. Lauraceae are an important component of tropical and subtropical forests and have major ecological and economic significance. Owing to lack of clear-cut morphological differences between genera and species, this family is an ideal case for testing the efficacy of DNA barcoding in the identification and discrimination of species and genera. The NJ tree based on the combined barcodes rbcL + matK + trnH–psbA + ITS revealed that *C. glanduliferum* was most related to *C. parthenoxylon* as a sister group owing to the previous studies did not include *C. septentrionale* (Liu et al. 2017). The complete chloroplast genome sequences of *C. septentrionale* will provide valuable bioinformatic information for future phylogenetic studies and the systematics of *Cinnamomum* (Figure 1).

**Figure 1.**
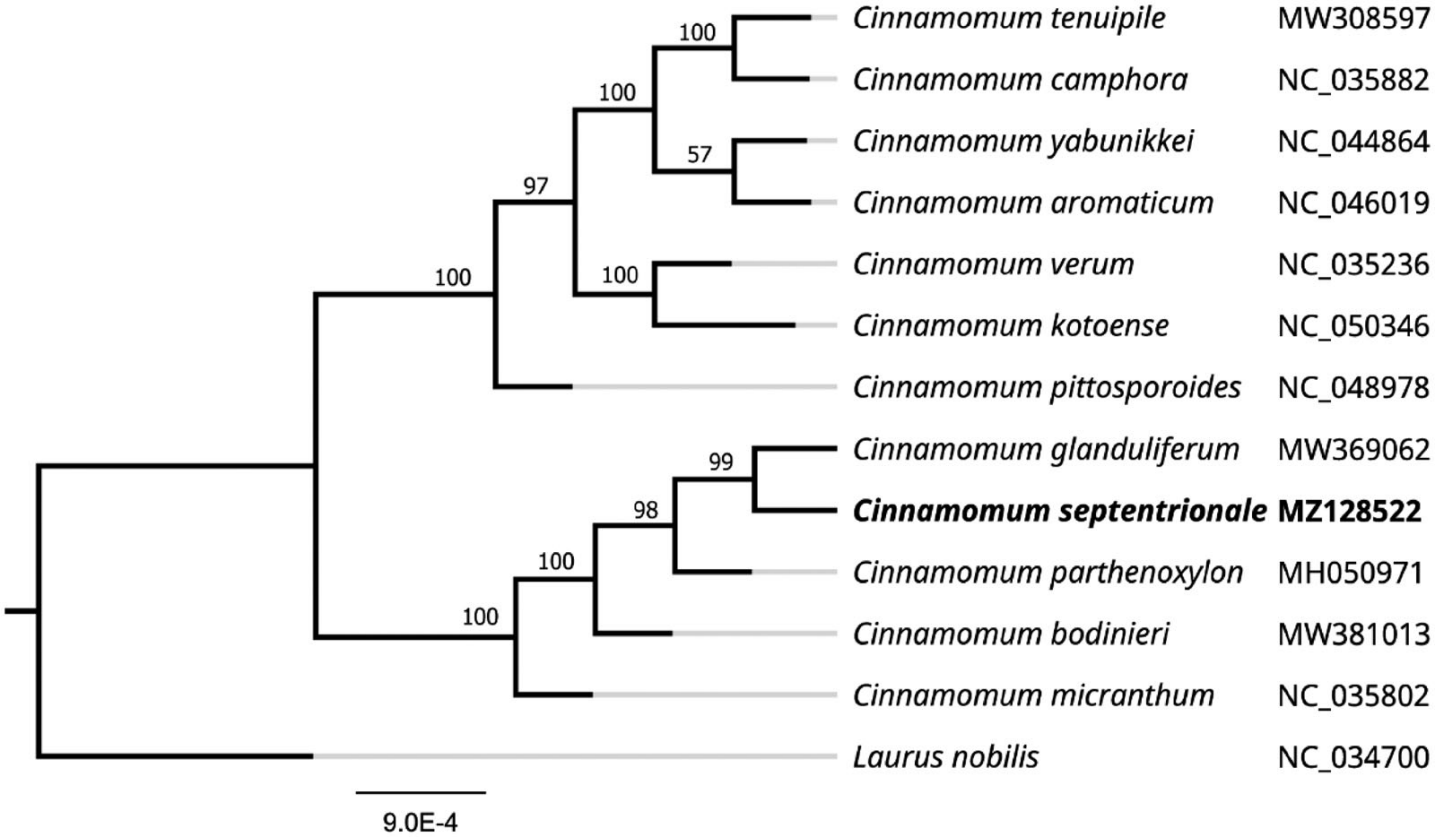
Maximum likelihood phylogenetic tree of *Cinnamomum septentrionale* and other related species based on the complete chloroplast genome sequence.

## Data Availability

The genome sequence data that support the findings of this study are openly available in GenBank of NCBI at (https://www.ncbi.nlm.nih.gov/) under the accession No. MZ128522. The associated BioProject, SRA, and Bio-Sample numbers are PRJNA737038, SRR14793489, and SAMN19678362 respectively.
